# Microsphere-Based Microfluidic Device for Plasma Separation and Potential Biochemistry Analysis Applications

**DOI:** 10.3390/mi12050487

**Published:** 2021-04-26

**Authors:** Hongyan Xu, Zhangying Wu, Jinan Deng, Jun Qiu, Ning Hu, Lihong Gao, Jun Yang

**Affiliations:** 1 Key Laboratory of Biorheological Science and Technology, Ministry of Education and Bioengineering College, Chongqing University, Chongqing 400030, China; 201819021046@cqu.edu.cn (H.X.); 202019131112@cqu.edu.cn (Z.W.); biojdeng@cqu.edu.cn (J.D.); huning@cqu.edu.cn (N.H.); 2 Department of Information, First Affiliated Hospital, Army Medical University, Chongqing 400042, China; 3 Chongqing Center for Drug Evaluation and Certification, Chongqing 401120, China

**Keywords:** microchip, microspheres stacking, plasma separation, concentration detection

## Abstract

The development of a simple, portable, and cost-effective plasma separation platform for blood biochemical analysis is of great interest in clinical diagnostics. We represent a plasma separation microfluidic device using microspheres with different sizes as the separation barrier. This plasma separation device, with 18 capillary microchannels, can extract about 3 μL of plasma from a 50 μL blood sample in about 55 min. The effects of evaporation and the microsphere barrier on the plasma biochemical analysis results were studied. Correction factors were applied to compensate for these two effects. The feasibility of the device in plasma biochemical analysis was validated with clinical blood samples.

## 1. Introduction

Blood biochemical tests are widely adopted for the screening of diseases in clinical diagnosis. However, red blood cells (RBCs) in the whole blood, with an intense red color, can interfere with test results [1,2]. Separation of the plasma from the whole blood sample is usually prerequired for an accurate determination of the components in a blood sample [1,3]. The centrifugation technique is a common method for plasma separation in a clinical test. However, this technique requires bulky and expensive equipment, limiting its applications in resource-limited settings [4]. Therefore, the development of a cost-effective point-of-care testing (POCT) device for plasma separation is highly desired.

Microfluidic techniques have shown great potential in the fabrication of POCT devices due to their low-cost, small size, low sample consumption and high-throughput analysis [5,6]. Numerous efforts have been made to design and fabricate microfluidic devices for plasma separation [3,7]. For example, paper-based microfluidic devices use porous cellulose material to remove RBCs from a whole blood sample [2,8,9,10]. Although these devices are simple and low-cost, the porous structure of the cellulose material is easily clogged by blood cells and retains proteins within its network structure [3]. Microstructures have been fabricated to perform plasma separation based on different mechanisms, such as digital microfluidics [11], cross-flow pillars [12,13], inertial force-based spiral channels [14,15,16,17], and the Zweifach–Fung separation technique [18,19]. With the elaborate design of microstructures, these devices can achieve a fast separation rate. However, the fabrication process of these microstructures is time-consuming, and precise control of the microfluid is also required for high separation efficiency. Previous research has shown that a separation system constructed by stacking microspheres in the microchannel can efficiently block blood cells and drive plasma forward by capillary force at the same time [20,21,22,23]. This method provides a simple way to selectively extract plasma from a whole blood sample without an external driving force. However, the separated plasma were directly applied in an immunoassay [22] and an agglutination test [23] assumed that the main components were perfectly preserved. The component concentrations in the plasma separated by those microfluidic devices have rarely been analyzed. It has been shown that the microstructures and surface properties of microfluidic devices can interact with the components in the plasma, which can lead to the component concentrations in the separated plasma being inconsistent with those of the “gold standard” of the centrifuge method [24,25].

In this work, we designed a microfluidic plasma separator with double-layer structure containing three types of microsphere layers. The formed microsphere barrier not only blocks RBCs but also allows the plasma to pass through by the capillary force without external driving forces. By controlling the stacking behavior of the microspheres and increasing the number of capillary channels connecting the microsphere barrier and the collection chamber, separation efficiency can be increased. The effects of water evaporation in the sample, induced by the open inlet and outlet of the device, and the microsphere barrier on the concentration variations of four components in the separated plasma were studied. Correction factors were also calculated for our device for the measurement of the four components in the separated plasma to make the results comparable to those with the traditional centrifuge method. Finally, the feasibility of using our device to separate clinical blood samples for a clinical biochemical test was studied.

## 2. Materials and Methods

### 2.1. Reagents and Materials

Phosphate buffered saline (PBS) was purchased from Solarbio Technology (Beijing, China). Protein blocking powder was obtained from Boster Biological Technology (Pleasanton, CA, USA). Photoresist (SU8 3050, SU8 2050) and the developer were provided by MicroChem (Newton, MA, USA). Polydimethylsiloxane (PDMS, Sylgard 184) was from Dow Corning (Midland, MI, USA). Microspheres with diameters of 10 µm, 20 µm and 100 µm were offered by Zhiyi Microsphere Technology (Suzhou, China).

### 2.2. Microchip Design and Fabrication

**Figure 1:**
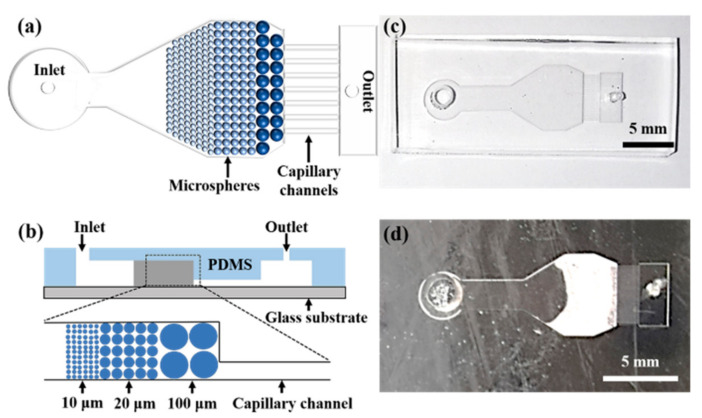
Schematic illustrations of the microfluidic chip of (**a**) the top view and (**b**) the cross section, and photos of the microchip (**c**) before and (**d**) after stacking microspheres.

The microfluidic plasma separator mainly contains four parts: the injection port, the microsphere stacking chamber, the straight capillary channels, and the collection chamber (Figure 1a,b). Microspheres were stacked in the microsphere stack chamber to block the RBCs and allow the plasma to pass through. The length and height of the microsphere stack chamber are 18.5 mm and 220 µm, respectively. The maximum width of the microsphere stacking chamber is 7.8 mm. There are 18 straight capillary channels connecting the microsphere stacking chamber and the collection chamber, which can provide a capillary force to promote the separated plasma toward the collection chamber with high throughput. The dimensions of each channel are 1.5 mm (l) × 100 µm (w) × 85 µm (h). The collection chamber is designed to collect the separated plasma, with the dimensions of 5.8 mm (l) × 2 mm (w) × 220 µm (h).

Because the height of the straight capillary channel is different from other parts in the device (Figure 1b), a double-layered master mold was made on a silicon wafer by the lithography technique. SU-8 3050 and SU-8 2050 were used to prepare the first layer with the height of 85 μm, and the second layer with the height of 220 µm, respectively. To prepare the PDMS pattern, the PDMS prepolymer and the curing agent were mixed at the weight ratio of 10:1 and degassed in the vacuum oven for 30 min to remove the air bubbles. The PDMS mixture was then poured on the SU-8 master mold and cured in an oven at 80 °C for 30 min. After curing, the PDMS pattern was peeled off from the master mold and the inlets and outlets were then punched. Then, the PDMS pattern was bonded to a pre-cleaned glass slide through oxygen plasma treatment (PDC-MG, Suzhou, China). The resulting plasma separation microchip is shown in Figure 1c.

### 2.3. Beads Stacking

A syringe pump (LSP10-1B, Lange, Baoding, China) was used to drive the microspheres into the stacking chamber for bead stacking. Protein blocking solutions containing 0.5 mg/mL silica microspheres with the diameter of 100 µm was first pumped into the microsphere stacking chamber at a flow rate of 600 µL/min. After all the microspheres of 100 µm were stacked at the entrance of the straight capillary tubes, the 20 µm and 10 µm microspheres were sequentially pumped into the microsphere stacking chamber at a flow rate of 600 µL/min. The volumes of 100 µm, 20 µm and 10 µm silica microspheres pumped into the chamber were 5 µL, 20 µL and 20 µL, respectively. After that, the chip was put on a hot plate at 75 °C for an hour to remove moisture, and was then left in a refrigerator (−20 °C) overnight to form the microsphere barrier to block the RBCs. The plasma separator containing the microsphere barrier is shown in Figure 1d. The length of the microsphere barrier is about 4.8 mm.

### 2.4. Plasma Separation

Human whole blood samples were collected from volunteers in Chongqing University Hospital. The blood samples were used for the experiment with the consent of the volunteers and Chongqing University Hospital. The blood samples used in the experiments were obtained from healthy volunteers aged between 18 and 40. Venous blood was used within 1 week after sampling and was stored in ethylenediaminetetraacetic acid (EDTA)-coated blood collection tubes at 4 °C. For the plasma separation, 50 µL blood sample was dropped into the inlet port and the plasma separation process was monitored under an optical microscope (IX73, Olympus, Tokyo, Japan) and recorded by a camera (DS126282, Canon, Tokyo, Japan).

### 2.5. Plasma Analysis

Assay kits for total protein (TP), albumin (ALB), glucose (GLU), and uric acid (UA), provided by Nanjing Institute of Biological Engineering (Nanjing, China), were used to measure the concentrations of components in the plasma samples. The component concentrations in the separated plasma were determined by the microplate reader (Bio Tek, Winooski, VT, USA). Plasma sample processing was performed according to the instruction by the supplier. For the measurement, 150 µL of the mixture of the plasma sample and the reaction reagent were pipetted into 96-well plate. The absorbance measurements were carried out at the corresponding wavelength with the microplate reader (Bio Tek, Winooski, VT, USA). For comparison, the concentrations of components in plasma samples prepared by the common centrifuge technique were assessed.

## 3. Results and Discussion

### 3.1. Microspheres Stacking

**Figure 2:**
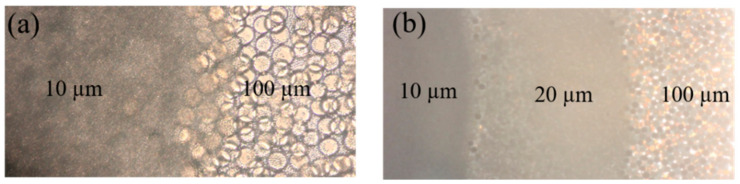
Optical microscopy images of (**a**) the stacking of 100 µm and 10 µm microspheres, and (**b**) stacking of microspheres with the size of 100 µm, 20 µm and 10 µm.

The major blood cells in blood are RBCs. Removing RBCs from the whole blood is important for plasma separation. The plate-shaped RBCs are 2 µm thick with a diameter of 8 µm [7]. To effectively block the RBCs and allow the plasma to pass through, the pore size of the stacking microspheres is a critical factor. Pores of too large size cannot block RBCs, while pores which are too small would be clogged by RBCs to prevent the movement of the plasma. Previous research has shown that the size of the microspheres should be controlled between 20 µm and 7.8 µm in order to prevent the movement of RBCs and allow the plasma to pass through at the same time [20]. However, if too few microspheres are stacked for the plasma separation, the amount of plasma separation collected is limited [21]. In order to achieve high-throughput plasma separation, three sizes of microspheres were used to create the barrier in this study. Large microspheres with a diameter of 100 µm are used first to prevent smaller microspheres from entering the separation channel, and which have limited contribution to the entire plasma separation process [20,23]. Plasma separation is realized by the 10 µm microspheres. However, we found that the small 10 µm microsphere could penetrate the 100 µm microsphere layer and move into the capillary channels if we directly pumped the 10 µm microsphere into the chamber, followed by the stacking of the 100 µm microsphere layer (Figure 2a). Therefore, we chose microspheres with the diameter of 20 µm as the transition layer between the two types of microspheres. After being dried on a hot plate, the microchip containing microsphere layers with clear boundaries is shown in Figure 2b.

### 3.2. Plasma Separation

**Figure 3:**
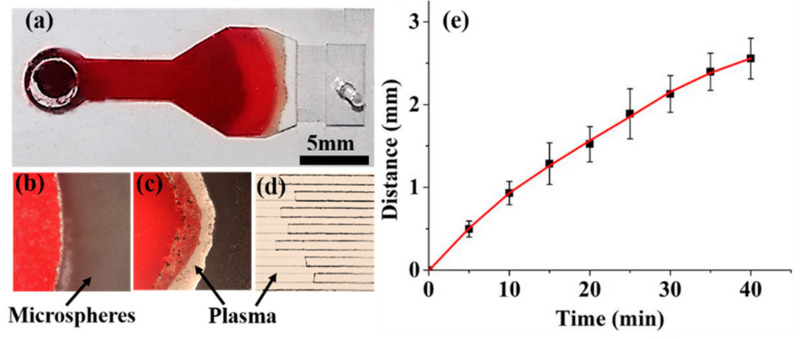
(**a**) Photo of the microchip during the plasma separation. Optical microscopy images of (**b**) the start separation of the plasma, (**c**) movement of the plasma within the microsphere barrier, and (**d**) flow of the plasma in the capillary microchannels. (**e**) The moving distance of the plasma as a function of time.

For the plasma separation, a 50 µL blood sample was dropped into the inlet port, allowing it to move into the microsphere stacking chamber for the plasma separation (Figure 3a). After the addition of the blood sample, the empty part of the microsphere stacking chamber quickly turned red in about 40 s, indicating that the blood sample can easily flow into the chamber without external forces. When it reached the microsphere barrier, the moving rate of the blood sample was reduced due to the large resistance of the barrier. After a while, a transparent band started to appear within the microsphere layers, which indicated that the plasma began to be separated from the blood sample (Figure 3b). The transparent plasma band gradually became clearer and enlarged (Figure 3c). After passing through the microsphere barrier, the transparent plasma quickly passed through the 18 capillary microchannels (Figure 3d, Video S1), and finally, accumulated in the collection chamber. The plasma in the capillary microchannels is transparent, suggesting that the capillary force added by the microsphere layers did not break the cell membrane of RBCs. The pores formed by the closely packed microspheres of 10 µm are too small to allow the RBCs to pass through the microsphere barrier, while the capillary force produced by the pores can promote the movement of plasma toward the collection chamber. In this way, the plasma can be separated from the blood sample. About 3 µL plasma was collected in 55 min with our device, which is acceptable for a POCT analysis [1,26,27]. Compared with the microbead-based device reported previously [21], which can only separate 350 nL plasma, the device in this study, with 18 capillary microchannels, shows a higher yield in plasma collection.

The movement of the front line of the separated plasma was recorded to study the kinetics of plasma separation. The zero point was set as the point where the plasma started to be separated from the blood sample. Figure 3e shows the moving distance of the midpoint of the front line as a function of time. The moving distance of the front line increases with the increase in time. The slope of the plot represents the moving rate of the separated plasma. The moving rate of the separated plasma gradually decreases with the increase of time. It took about 40 min for the plasma to pass through the microsphere barrier and the capillary channels. As the amount of the plasma separated increases, the blood cells accumulated near the edge of the microsphere barrier will increase the viscosity of the remaining blood. Therefore, the moving rate of the plasma gradually slows down, which is also reported by previous studies [7,23,28]. About 3 µL plasma can be collected in 55 min with this device.

### 3.3. Plasma Analysis

**Figure 4:**
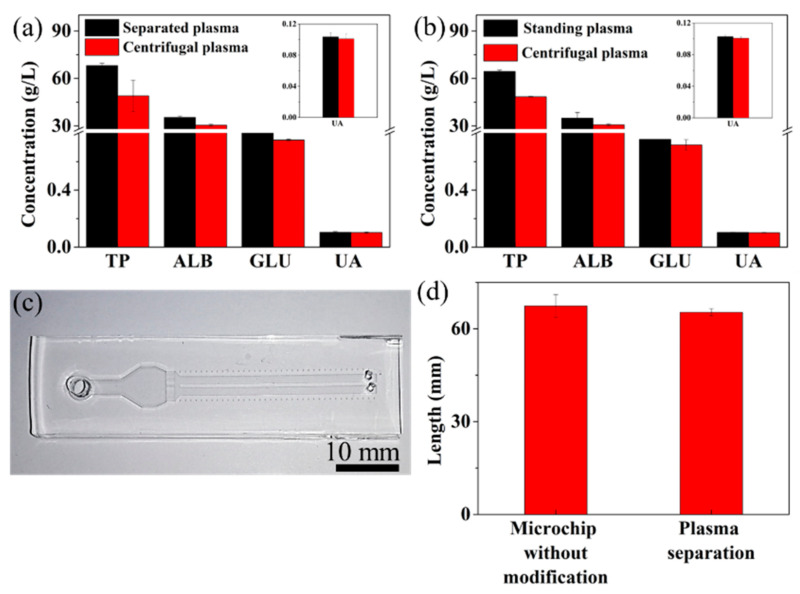
(**a**) Component concentrations of the separated and centrifugal plasma samples; inset is the UA concentration of separated and centrifugal plasma samples. (**b**) Absorbance of standing and centrifugal plasma; inset is the UA concentration of separated and centrifugal plasma samples. (**c**) The photo of the microdevice for the study of the effect of microsphere barrier on the measurement results. (**d**) The length of water separated from the device.

Proteins and various small molecules in the blood plasma are necessary for sustaining health, and they are usually adopted as biomarkers for the clinical diagnosis of diseases [1,3]. For example, glucose concentrations in the plasma are usually considered as the “gold standard” in the clinical diagnosis of diabetes [29,30]. In order to prove the feasibility of our device in the application of plasma analysis, we employed four assay kits to assess the component concentrations in the separated plasma by our device. The component concentrations in the plasma prepared by traditional centrifuge technique were used as comparison. Figure 4a shows concentrations of four components—TP, ALB, GLU, and UA—in the separated plasma by our device, and centrifugal plasma by centrifuging. The concentrations of the four components in the separated plasma show different degrees of increase compared with those in the centrifugal plasma, which show about 19.133 g/L increase for TP, 4.85 g/L increase for ALB, 0.0489 g/L increase for GLU, and 0.004 g/L increase for UA, respectively (Figure 4a).

Considering the separation time (~55 min) and the dried protein blocking coating on the surface of the microspheres, we hypothesize that the evaporation of water in the sample from the open inlet and outlet of the device, and the microsphere barrier, are the main reasons that lead to the increased component concentration for the separated plasma. Standard plasma samples were used to prove our hypothesis. For the study of the influence of evaporation, a 50 µL centrifugal plasma sample was dropped into the inlet of the device and collected from the collection chamber after 55 min, before the absorbance measurement, which is the same length of time for the plasma separation process. Compared with centrifugal plasma without standing, the standing plasma shows a stronger absorbance for the four components (Figure 4b) which lead to concentration increases of 15.95 ± 0.92 g/L for TP, 4.23 ± 2.9 g/L for ALB, 0.038 ± 0.007 g/L for GLU, and 0.002 ± 3.8 × 10−5 g/L for UA, respectively. This suggests that evaporation of the water in the plasma during the separation process can lead to an increased concentration of the components in the plasma. Then, we designed two straight channels with a scale, instead of the collection chamber connecting the capillary channels (Figure 4c), to study the influence of the microsphere barrier on the measurement results. The straight channel is 2 mm wide, 0.22 mm high and 37 mm long. The straight channel allows us to precisely measure the volume of the sample separated from the microsphere barrier. Deionized water was used to perform this experiment. After the addition of 50 µL deionized water into the device for about 10 min, the length of water going out from our device was 65.3 mm, while it was 67.4 mm for the device containing the barrier without the protein blocking coating (Figure 4d). The stacked silica microspheres in the chamber created a large surface area for the sample to make contact with. The dried hydrophilic protein blocking layer coated on the surface of microspheres can uptake some amount of water in the sample, which can lead to increased measurement results of plasma components. The volume difference between these two devices is 0.924 μL, which can lead to concentration increases of 0.919 g/L for TP, 0.571 g/L for ALB, 0.0129 g/L for GLU, and 0.0018 g/L for UA, respectively. Therefore, the effects of evaporation and microsphere barrier lead to concentration increases of 16.87g/L for TP, 4.801 g/L for ALB, 0.051 g/L for GLU, and 0.0039 g/L for UA, which are similar to the concentration differences between the separated and centrifugal plasma samples. The slight difference is due to the different affinities between the protein blocking coating and the components, which lead to different amounts of component adhered within the microsphere barrier. In order to obtain measurement results that were comparable to those in the centrifugal sample, correction factors were calculated for the four components measured in this study (Table 1).

### 3.4. Clinical Validation

**Figure 5:**
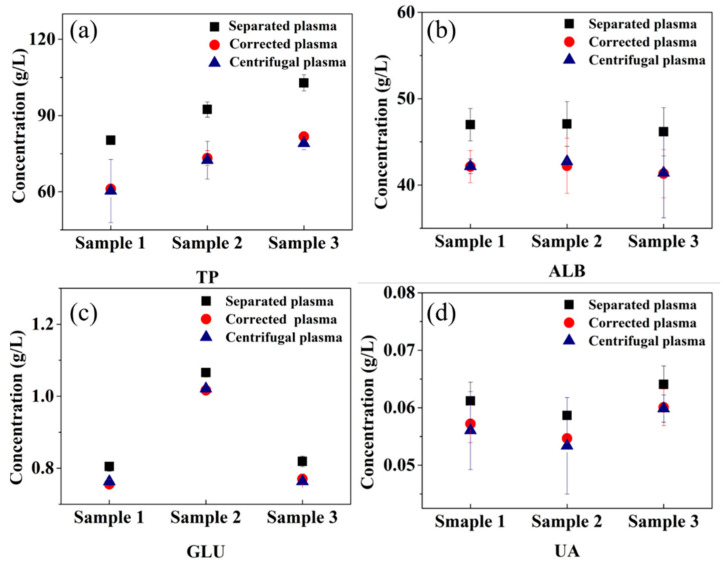
Component concentrations of clinical samples: (**a**) TP, (**b**) ALB, (**c**) GLU, and (**d**) UA.

In order to assess the validity of the correction factors, we used blood samples from three volunteers to measure the component concentrations in the plasma separated by our device. In the process of plasma separation of each human blood sample, no hemolysis occurred. The four component concentrations in all three separated samples are higher than those in centrifugal samples before correction (Figure 5). The concentration differences for TP, ALB, GLU, and UA between the separated and the centrifugal samples are 21.203 ± 2.205 g/L, 4.978 ± 0.765 g/L, 0.043 ± 0.012 g/L, and 0.005 ± 0.001 g/L respectively. After correction, the component concentrations in separated sample show an obvious decrease and move toward the concentrations of centrifugal ones. The concentration differences of components between the corrected and centrifugal samples are 1.408 ±1.056 g/L, 0.168 ± 0.765 g/L, 0.006 ± 0.012 g/L, and 0.001 ± 0.001 g/L for TP, ALB, GLU, UA, respectively, which is acceptable for plasma analysis.

## 4. Conclusions

We present a simple plasma separation device containing three layers of microspheres with different sizes as the separation barrier. It takes about 55 min to extract 3 μL of plasma from a 50 μL blood sample with this device. The evaporation and the absorption of water by the protein blocking coating of the microspheres during the separation process are the main causes of increased component concentrations in the plasma. Correction factors are applied to the device to eliminate those two factors. The feasibility of this device for clinical biochemical testing applications is validated with clinical blood samples for the measurement of TP, ALB, GLU, and UA in the separated plasma samples. The concentration differences between the separated plasma with our device and the centrifugal plasma after correction are 1.408 ± 1.056 g/L for TP, 0.168 ± 0.765 g/L for ALB, 0.006 ± 0.012 g/L for GLU, and 0.001 ± 0.001 g/L for UA, respectively, which are acceptable for POC analysis. This cost-effective, portable and external force-free plasma separation microchip shows great potential in POC analysis of blood biochemistry.

## Figures and Tables

**Table 1:** Concentration correction value for the plasma component measurement.

Types of Components in Plasma	Correction Factor (g/L)	Standard Deviation
TP	19.133	8.58
ALB	4.854	0.199
GLU	0.049	0.004
UA	0.004	0.001

## Supplementary Materials

The following are available online at https://www.mdpi.com/article/10.3390/mi12050487/s1, Video S1: the separated plasma passes through the capillary channels.
